# Systematic training in focused cardiopulmonary ultrasound affects decision-making in the prehospital setting – two case reports

**DOI:** 10.1186/1757-7241-22-29

**Published:** 2014-05-01

**Authors:** Louise Kollander Jakobsen, Morten Thingemann Bøtker, Lars Peter Lawrence, Erik Sloth, Lars Knudsen

**Affiliations:** 1 Department of Anaesthesiology, Thy-Mors Hospital, Northern Denmark Region, Thisted, Denmark; 2 Research Department, Prehospital Emergency Medical Services, Central Denmark Region, Aarhus, Denmark; 3 Research Center for Emergency Medicine, Aarhus University Hospital, Aarhus, Denmark; 4 Helicopter Emergency Medical Services, Central and Northern Denmark Region, Karup, Denmark; 5 Department of Anesthesiology and Intensive Care, Aarhus University Hospital, Aarhus, Denmark

**Keywords:** Focused ultrasound, Prehospital, Echocardiography, Pulmonary embolism, Pericardial effusion, Aortic dissection, Dyspnea

## Abstract

**Abstact:**

We present two cases from the Helicopter Emergency Medical Services (HEMS) in Denmark, in which prehospital physicians trained in cardiac ultrasound (FATE) disclosed significant pathology that induced a radical change for the critical patient’s course.

## Background

The need for fast decision-making in the prehospital setting is evident. Often, decisions are based solely on medical history and a perfunctory physical examination. With the development of new handheld ultrasonography devices, it is now possible to perform ultrasonography out-of-hospital. Furthermore, ultrasound in the prehospital setting is pointed out to be one of the five high priority research areas in prehospital medicine [1].

However, the use of ultrasonography in the prehospital setting is controversial – should we stay-and-play or scoop-and-run?

In order for ultrasonography to improve patient outcome, a balance between time consumption and advantage gained is needed and it is imperial to assess, which patients benefit from examinations that potentially prolong on-scene time, and which do not [2]. Recent case reports show how prehospital focused cardiac ultrasound can be used to guide triage and treatment in prehospital shock management [3], e.g. life-threatening aortic aneurism and pulmonary embolism [4,5].

During fall 2012, physicians staffing the Helicopter Emergency Medical Services (HEMS) in the Central Denmark Region completed a systematical educational program in Focused Assessed Transthoracic Echocardiography (FATE) [6]. This comprised of e-learning, one-day hands on FATE course and ten supervised real-time examination in patients admitted to hospital.

In the following, we present two cases from the HEMS after this educational program in which focused ultrasonography significantly contributed to prehospital decision-making.

## Cases

### Case 1

A 55-year-old otherwise healthy male developed sudden shortness of breath during a bike ride, making it impossible to continue the trip. The Emergency Medical Communication Center (EMCC) was contacted and a local ambulance and a Helicopter Emergency Medical Service, staffed with an emergency physician, were dispatched. A standard prehospital medical evaluation including 12 lead ECG was done. Vital parameters followed an unspecific pattern often seen in patients with dyspnea - respiration frequency 25/min, saturation of 93–94% without oxygen supply, heart rate 100–110 bpm and blood pressure of 115/60 mmHg. Physical examination revealed no obvious pathology. ECG showed normal sinus rhythm.

**Figure 1:**
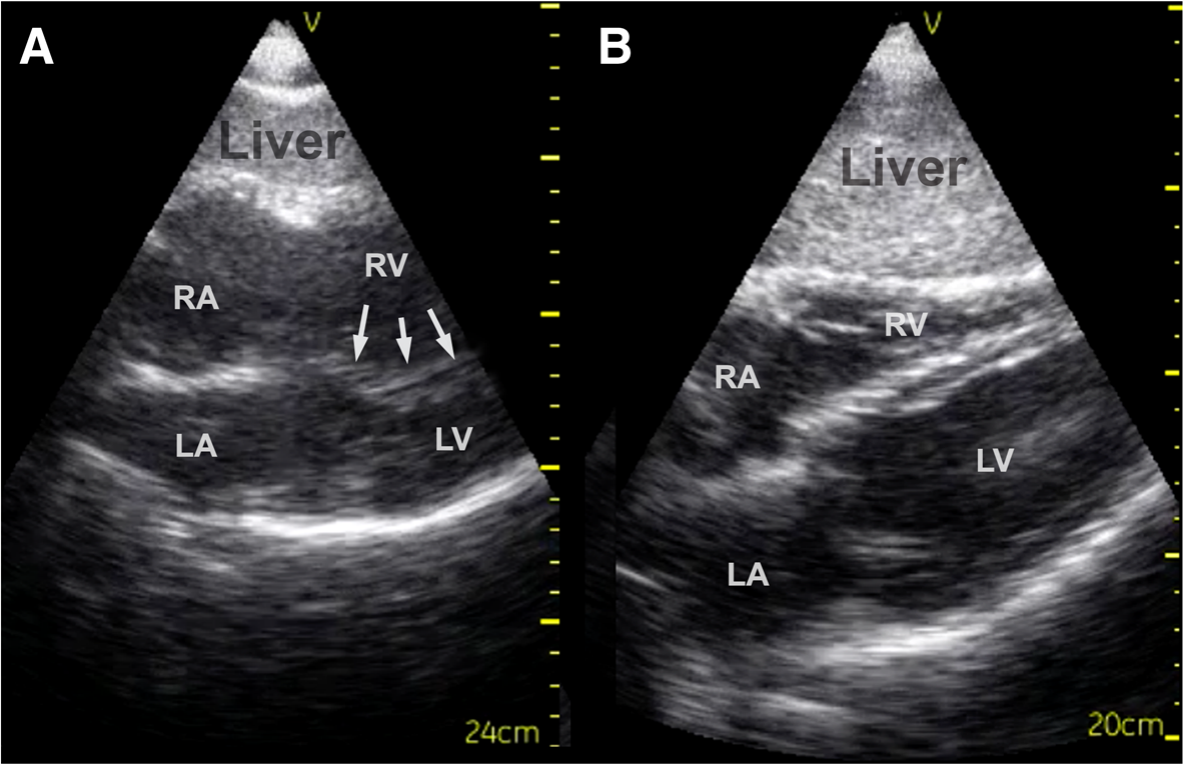
**Subcostal 4-chamber view – pulmonary embolism and normal. A**: Subcostal 4-chamber view - pulmonary embolism. Notice the dilated right ventricle and bulging of the septum into the left ventricle in the pathological echocardiogram because of the increased pressure in the right ventricle (arrows). The pattern of the septum can often be seen even when image-quality is low. **B**: Subcostal 4-chamber view – normal. RA = Right Atrium, RV = Right Ventricle, LA = Left Atrium, LV = Left Ventricle.

**Figure 2:**
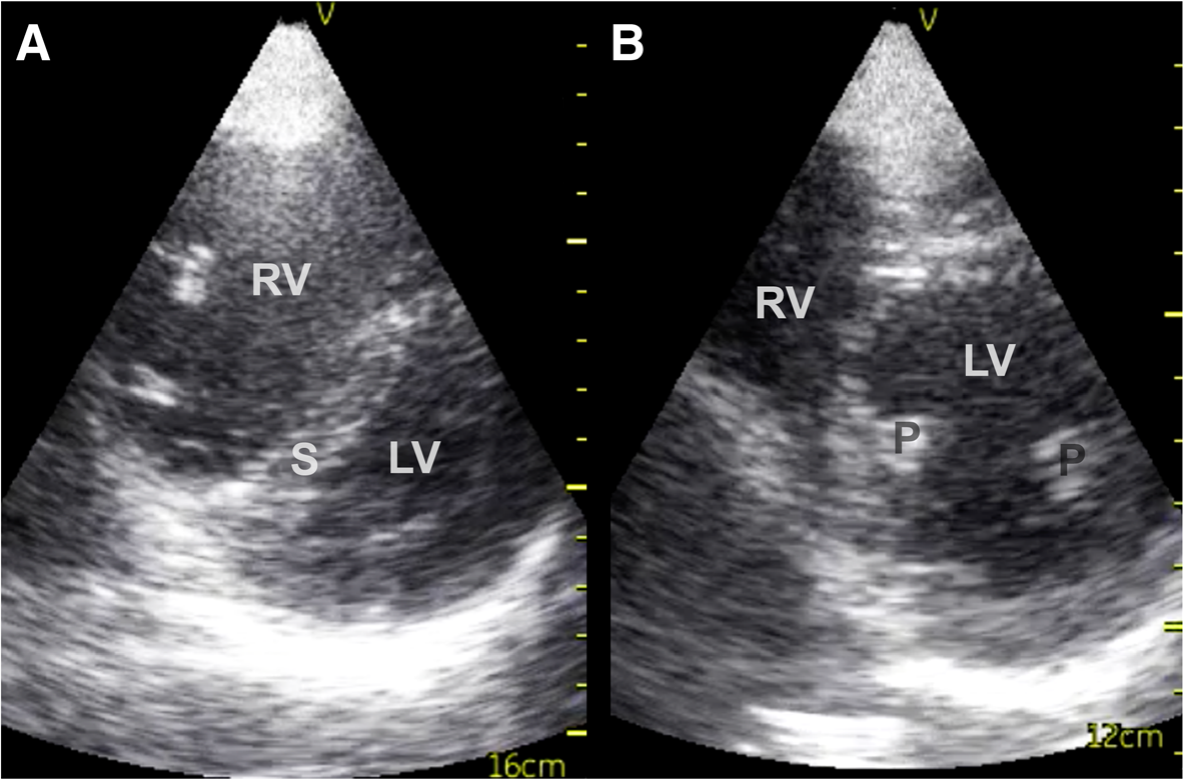
**Parasternal short axis view – pulmonary embolism and normal. A**: Parasternal short axis view – pulmonary embolism. Notice the bulging of the septum into a typical D-shaped left ventricle. **B**: Parasternal short axis view - normal. RV = Right Ventricle, LV = Left Ventricle, S = Septum, P = Papillary muscles.

FATE examination disclosed a dilated right ventricle with characteristic septum shift towards the left ventricle in both subcostal and apical 4 chambers views and a D-shaped compromised left ventricle in systole in parasternal cross-section view (Figures 1 and 2). These are typical signs of a pulmonary embolism that leads to hemodynamic compromise. Based on the ultrasound examination, a radical change in the course for the patient followed. Prehospital heparinization (10.000 units of standard unfractionated heparin), and triage directly to specialized cardiologic department for thrombolysis was initiated. Shortly after admission to the hospital, computed tomography confirmed the diagnosis. Thrombolytic therapy was initiated within 2 hours after the emergency call to the EMCC. Four hours after the emergency call, the patient was without subjective symptoms and had normalized vital parameters. Long-term anticoagulation therapy was initiated. The patient recovered completely.

### Case 2

A 60-year-old, otherwise healthy male, developed left hemiparesis and the EMCC was contacted. As the patient was a candidate for thrombolysis because of suspected ischemic stroke, local ambulance and HEMS staffed with emergency physician were dispatched. On arrival of the HEMS, the medical history revealed a slow development of mild chest pain preceding the hemiparesis. Vital parameters were not specific; respiration frequency 20/min, saturation 95% without oxygen supply, heart rate 90 bpm and blood pressure 120/70 mmHg. Physical examination showed slight aphasia and partially paresis of the left arm. ECG showed normal sinus rhythm.

**Figure 3:**
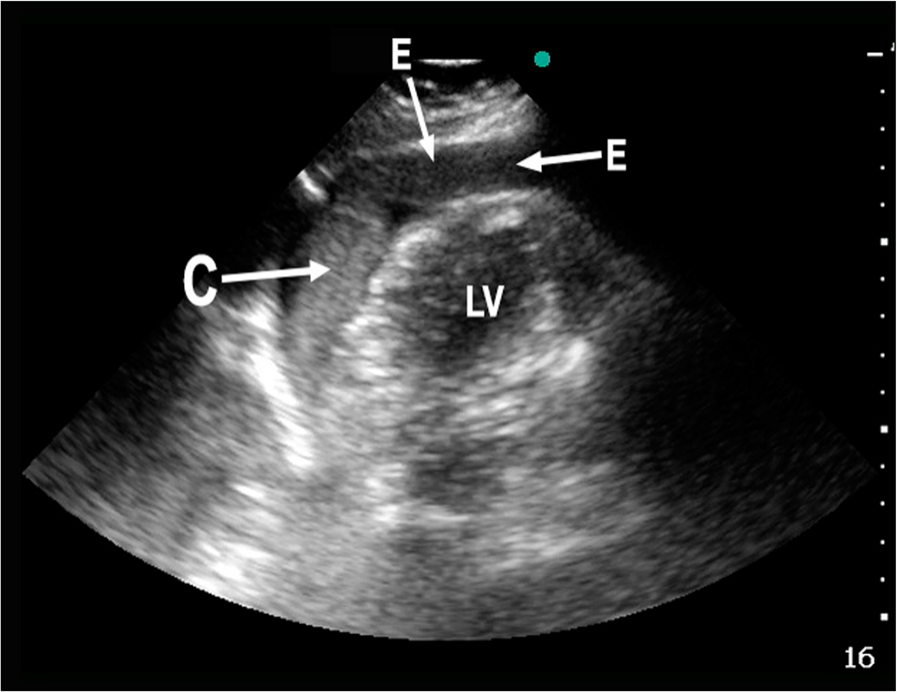
**Reversed apical 4-chamber view – pericardial effusion.** Pathological reversed apical 4-chamber view (the transducer is rotated 180 degrees compared to the classical apical 4-chamber view) from patient with aortic dissection and pericardial effusion. E = Effusion (blood), C = Clot, LV = Left Ventricle.

Because of the diverging symptoms, a FATE examination was done. Somewhat surprisingly, this disclosed a large pericardial effusion (Figure 3). Based on this finding, aortic dissection was suspected. Because of an obvious need to reduce time to definite treatment, no further efforts were done to visualize the thoracic aorta. Again, based on the ultrasound findings, a radical change in the course for the patient followed. Instead of referral for thrombolysis at a nearby hospital, the patient was admitted to an invasive heart center 130 km away. Echocardiography and computed tomography of the thorax confirmed a dilated aorta that had perforated into the pericardium and dissected up into the right carotid artery. During the 25 minutes transportation time with HEMS, the receiving hospital was able to prepare an operating theater. After supplemental imaging, emergency surgery with replacement of the disrupted part of aorta was performed. The patient was discharged with minimal cognitive dysfunction due to perioperative cerebral ischemia, but with no physical sequelae.

## Discussion

In these cases, focused ultrasonography significantly contributed to decision-making in the prehospital setting. Ultrasonography changed medical treatment and induced a radical change in the patient’s disposition by reducing time to definite treatment as the patients were transported directly to a facility with the necessary resources. Further, because of the prehospital diagnosis, the receiving units were alerted and treatment was initiated upon arrival without delay.

The two cases deal with two of the top five research priorities in physician-provided pre-hospital critical care [1]; systematic training of prehospital personnel and the use of prehospital ultrasound. Based on these cases, prehospital ultrasound can positively affect patient management and the patient pathway. The cases provide additional evidence regarding the benefit of prehospital focused ultrasound in patients with time critical diagnosis. Others have shown benefit in cardiac arrest patients, patients with chest pain and patients with dyspnea. In patients with dyspnea, a recent case has shown, that prehospital lung ultrasound may also be valuable [7]. Cases of lifesaving changes in patient-course based on focused cardiac ultrasound in trauma patients have also been reported [8-10]. However, ultrasound beams do not save lives, the right decisions do. Thus, it is important that focused ultrasonography is not used as a replacement for routine medical history and physical examination. It should be used as a supplement to aid the physician in making the right decision.

This case presentation is only the first step to providing evidence for using focused ultrasound in the prehospital setting. Randomized controlled studies evaluating the impact of this intervention in the field on patient outcomes are needed [11] and international collaboration is highly preferable.

Routine clinical use and clinical research is only achievable with systematic high-quality education. Furthermore,“close at hand” access to ultrasound devices is a perquisite. The Nordic countries have widespread prehospital units staffed by anesthesiologists, a leading position in focused ultrasound education and a broad ultrasound availability, altogether giving ideal foundation for examining the area further.

## Conclusion

In these cases, systematic training in focused cardiac ultrasonography significantly contributed to decision-making in the prehospital setting.

Descriptive, hypothesis-generating studies and then outcome-studies on routine clinical use are needed. In order to pursue this, systematic high-quality education is a precondition.

## Consent

Written informed consent was obtained from the patients for publication of this case report and accompanying images. A copy of the written consent is available for review by the Editor-in-Chief of this journal.

## Competing interest

The authors declare that they have no competing interests.

## Authors’ contributions

MTB, LK, LPL and ES conceived the idea of these case reports. They have all been involved in drafting the manuscript and revising it critically for intellectual content. LKJ has been involved in drafting the manuscript, revising it critically for intellectual content and has conducted the literature review. MTB planned and conducted the educational program necessary to implement the examination and LK and LPL have collected cases 1 and 2, respectively. All authors have read and approved the final manuscript.
